# MEDICAL COMPLICATIONS AND INJURY LEADING TO EMERGENCY DEPARTMENT USE AMONG OLDER ADULTS

**DOI:** 10.1093/geroni/igz038.1787

**Published:** 2019-11-08

**Authors:** Mary W Carter, Bo Kyum Yang, Cyrus Y Engineer

**Affiliations:** Towson University, Towson, Maryland, United States

## Abstract

Medical injury consistently ranks among the most expensive hospital stay diagnoses and represents a frequent cause of hospital readmission. Although older adults are at greater risk of medical injury, in part, because of greater incidence of comorbidity and increased medical complexity, little is known about the burden of medical injury leading to ED use or the costs and outcomes associated with these events among older adults. In response, this study used nationally representative data from the 2014 Nationwide Emergency Department Survey to examine the epidemiology of older adult ED-visits for medical injury. Principal diagnosis codes were grouped using AHRQ’s Clinical Classification Software to identify medical injury-related visits. Results indicated that in 2014, 506,466 ED-visits for medical injuries occurred, comprising 2% of all older adult ED-visits. Leading causes of medical injury included malfunction of device, implant and grafts (24%); infection and inflammation of internal prosthetic device, implant, and graft (16%), and other complications of surgical and medical procedures (15%). Risk factors for medical injury included being male, Medicaid as primary payor, and number of chronic conditions. Multinominal logistic regression and multivariate regression results indicate that Medical injury-related ED visits were associated with higher hospitalization risk (RRR=2.08, p<0.000), 27% longer hospital stays, and 24% higher total charges relative to non-medical injury related visits. However, medical injury was not associated with risk of death after adjustment. Study findings suggest that ED-visits for medical injury occur frequently among older adults and are associated with significant burden and cost.

